# New Insights on Plant Salt Tolerance Mechanisms and Their Potential Use for Breeding

**DOI:** 10.3389/fpls.2016.01787

**Published:** 2016-11-29

**Authors:** Moez Hanin, Chantal Ebel, Mariama Ngom, Laurent Laplaze, Khaled Masmoudi

**Affiliations:** ^1^Laboratoire de Biotechnologie et Amélioration des Plantes, Centre de Biotechnologie de SfaxSfax, Tunisia; ^2^Institut Supérieur de Biotechnologie, Université de SfaxSfax, Tunisia; ^3^Laboratoire mixte international Adaptation des Plantes et microorganismes associés aux Stress EnvironnementauxDakar, Senegal; ^4^Laboratoire Commun de Microbiologie, Institut de Recherche pour le Développement/Institut Sénégalais de Recherches Agricoles/Université Cheikh Anta DiopDakar, Senegal; ^5^Institut de Recherche pour le Développement, Unités Mixtes de Recherche, Diversité, Adaptation, Développement des Plantes (DIADE), MontpellierFrance; ^6^Department of Aridland, College of Food and Agriculture, United Arab Emirates UniversityAl Ain, UAE

**Keywords:** salinity, tolerance mechanisms, transport of sodium, detoxification pathways, beneficial soil microorganisms, engineering of plant salinity tolerance

## Abstract

Soil salinization is a major threat to agriculture in arid and semi-arid regions, where water scarcity and inadequate drainage of irrigated lands severely reduce crop yield. Salt accumulation inhibits plant growth and reduces the ability to uptake water and nutrients, leading to osmotic or water-deficit stress. Salt is also causing injury of the young photosynthetic leaves and acceleration of their senescence, as the Na^+^ cation is toxic when accumulating in cell cytosol resulting in ionic imbalance and toxicity of transpiring leaves. To cope with salt stress, plants have evolved mainly two types of tolerance mechanisms based on either limiting the entry of salt by the roots, or controlling its concentration and distribution. Understanding the overall control of Na^+^ accumulation and functional studies of genes involved in transport processes, will provide a new opportunity to improve the salinity tolerance of plants relevant to food security in arid regions. A better understanding of these tolerance mechanisms can be used to breed crops with improved yield performance under salinity stress. Moreover, associations of cultures with nitrogen-fixing bacteria and arbuscular mycorrhizal fungi could serve as an alternative and sustainable strategy to increase crop yields in salt-affected fields.

## Introduction

It is expected that world population will continue to grow and exceed nine billion by 2050 (Department of Economic and Social affairs of the United Nations, 2015^1^. Therefore, the global food production must increase substantially to ensure food security for the growing population. However, food production is seriously threatened by various environmental factors and soil salinity is one of the major stresses adversely affecting plant growth and crop productivity, especially in arid and semi-arid regions. Worryingly, these regions continue to expand and they represent today 40% of the world’s land surface where two billion people are living, mostly in developing countries (UNEP, 1992; Flowers and Yeo, 1995). As a result, more irrigation with brackish water is unavoidable and salinization becomes a serious agricultural concern worldwide. Therefore, engineering crops with enhanced salt stress tolerance traits is one of the most important challenges for modern agriculture.

This review focuses on the state of the art regarding the effect of soil salinity on plant growth and gives an overview of mechanisms controlling salt stress tolerance from sensing and signaling to gene expression and adaptive plant responses. Such knowledge is primordial for setting molecular approaches to enhance plant salinity tolerance. We will also discuss the potential of beneficial soil microorganisms in providing sustainable alternatives for improving crop production in saline soils.

## Salinization in Arid and Semi-Arid Region and the Problem of Land Degradation

According to standard definition, saline soils are those which have an electrical conductivity (EC) of the saturation soil-paste extract of more than 4 dS/m at 25°C, which corresponds to approximately 40 mM NaCl and generates an osmotic pressure of approximately 0.2 MPa (Munns and Tester, 2008; USDA-ARS, 2008). When grown on soils with an EC value above 4, crops significantly reduce their yield. Salts may include chlorides, sulfates, carbonates and bicarbonates of sodium, potassium, magnesium, and calcium, the diverse ionic composition of salt-affected soils results in a wide range of physiochemical properties. In the case of saline-sodic soils growth is hindered by a combination of high alkalinity, high Na^+^, and high salt concentration (Eynard et al., 2005). In this regard, it is important to distinguish between soil salinization and soil sodicity.

Soil salinization is referred as the accumulation of soluble salts in the soils (Bockheim and Gennadiyev, 2000). This is particularly favored by arid and semi-arid climates with evapotranspiration volumes being greater than precipitation volumes along the year.

Soil sodicity is a term given to the amount of Na^+^ detained in the soil. High sodicity (more than 5% of Na^+^ of the overall cation content) causes clay to swell excessively when wet, hence limiting severely air and water movements and resulting in poor drainage.

Salts may arise naturally in subsoil (primary salinization) or maybe be introduced (secondary salinization) by soil amendments, inorganic fertilizers, and most importantly irrigation with brackish water (Carillo et al., 2011). As a result, the total area of salt-affected lands in the world is estimated at more than 800 million hectares (ha), which account for more than 6% of the world’s total land area. Of the current 230 million ha of irrigated land, 45 million ha (19.5%) have been already damaged by salt (FAO, 2016).

As NaCl is the most soluble and widespread salt, plants have evolved mechanisms to tolerate/exclude it while allowing acquisition of other nutrients available at low concentrations, such as phosphate, potassium, and nitrate.

## Impact of Soil Salinization on Plant Growth and Survival

High soil salinity impacts the growth of numerous plant species especially glycophytes (salt-sensitive compared to salt-tolerant halophytes species), wherein fall major crops. Salt stress tolerance level varies from one species to another and, for cereal crops, bread wheat is a moderately salt-tolerant crop (Maas and Hoffman, 1977). In field conditions, the wheat crop yield will be reduced in the presence of 100 mM NaCl (10 dS/m), whereas rice cannot survive up to maturity under such conditions. Barley (*Hordeum vulgare*), the most tolerant cereal, can tolerate up to 250 mM NaCl (equivalent to 50% seawater), beyond which the survival rates drop drastically. Other cereals, such as durum wheat (*Triticum turgidum* ssp.), maize (*Zea mays*), and sorghum (*Sorghum bicolor*) are less tolerant to salinity (Maas and Hoffman, 1977).

The reduction in plant growth following salt exposure is due to two phases, osmotic stress and ionic toxicity (Munns and Tester, 2008). Upon a salt stress, the first phase is a rapid response to an increase in the osmotic pressure of the soil solution, whereas the second one is a slower response and takes place after the accumulation of Na^+^ in photosynthetic tissues. Although they can be clearly identified in most plants and under various salt stress conditions, these two phases are not obvious under high salinity or in the case of Na-hypersensitive plant species such as rice (Munns and Tester, 2008).

It is noteworthy that the overall leaf/shoot development is more sensitive to salinity than root growth and it is assumed that less expanded leaves would decrease the water use by the plant, hence allowing it to conserve soil moisture and prevent a further rise in the salt concentration in the soil. Knowledge about the mechanism by which leaf growth and shoot development are down-regulated under salt stress is relatively scarce, but within days following a salt stress, there is evidence for the involvement, in this inhibitory mechanism, of long distance signals consisting of mainly hormones, as it was previously reported in barley (Munns et al., 2000).

Moreover, salt may affect plant growth indirectly by decreasing the rate of photosynthesis and stomatal conductance (Brugnoli and Lauteri, 1991). Decrease in stomatal aperture is considered as the most dramatic response that occurs soon after plant exposure to salinity owing to the osmotic effect of salt outside the roots (Munns and Tester, 2008).

Stomata are the main structures responsible for gas exchange control, and salt stress affects not only stomatal opening but also their size and density, resulting in a decrease in stomatal conductance. Consequently, rates of transpiration (i.e., water loss) and photosynthesis (CO_2_ uptake) are also reduced. Indeed, in cotton plants submitted to salt stress treatments, a substantial reduction in photosynthesis has been associated with a decrease in total chlorophyll content and distortion in chlorophyll ultrastructures (Zhang L. et al., 2014). It has been also evoked that sink to source feedback inhibition would moderate the rate of photosynthesis to match the reduced demand arising from growth inhibition (Paul and Foyer, 2001).

On the other hand, the reduced rate of photosynthesis increases the formation of reactive oxygen species (ROS), and enzymatic antioxidant activities such as superoxide dismutase (SOD), catalase (CAT), and various peroxidases (Apel and Hirt, 2004; Foyer and Noctor, 2005; Logan, 2005). These functionally inter-related enzymes act in a coordinated manner to ensure a balance between the rate of formation and removal of ROS. However, previous studies in *Arabidopsis* have suggested that the mode of coordination between different components of the ROS removal network is complex, since ROS are also used by plants as signaling molecules controlling different processes such as growth, development, and stress responses (Mittler et al., 2004).

## Salt Tolerance Mechanisms in Plants

### Sensing and Signaling to Regulate Plant Salt Stress Response

To tolerate salt in soil solution, plants deploy a variety of traits to control cell function and development that relies on signal perception, signal integration, and processing. The development of high-throughput sequencing technologies during the last few years has generated huge quantity of data and led to the discovery of several signaling molecules. Among them, are those involved in the activation of signaling pathways that can enhance plant ability to tolerate salt stress. ROS, despite their potential toxicity, have the advantage of being versatile signaling molecules with regard to their properties and mobility within cells. The mitogen-activated protein kinase (MAPK) can trigger plant response to biotic and abiotic stresses by activating the antioxidant enzymes. Many different MAPKs cascades are activated upon ROS accumulation. These include the ROS-responsive MAPKKK, MEKK1, MPK4, and MPK6 (Xing et al., 2008; Jammes et al., 2009). ROS signaling is tightly linked to cellular homeostasis and highly integrated with hormonal signaling networks, allowing plants to regulate development processes, as well as adaptive responses to environmental constraints (Miller et al., 2010). Increased generation and accumulation of ROS such as superoxide (O_2_^-^), hydrogen peroxide (H_2_O_2_), and nitric oxide (NO) cause oxidative damages in the apoplastic compartment and lipid peroxidation of cellular membranes, and have an extensive impact on ion homeostasis by interfering with ion fluxes (Baier et al., 2005). Excess of ROS levels are particularly scavenged by antioxidant metabolites such as ascorbate, glutathione, tocopherols, and by ROS detoxifying enzymes such as SOD, ascorbate peroxidase (APX), and CAT. Moreover, increased ROS levels can cause salicylic acid accumulation contributing to plant defense, cell death, and induced stomatal closure (Khokon et al., 2011). Recent advance in considering the important role of ROS in plant salt responses was the discovery of a coupled function of plastid heme oxygenases and ROS production in salt acclimation (Xie et al., 2011). These findings strongly suggest involvement of the chloroplast to nucleus signaling pathway in plant salt adaptation. In depth study on cross-species expression of a SUMO conjugating enzyme has provided considerable insight into the links between ROS, ABA (abscisic acid) dependent signaling and the sumoylation pathways in plant salt and drought tolerance (Karan and Subudhi, 2012).

**FIGURE 1:**
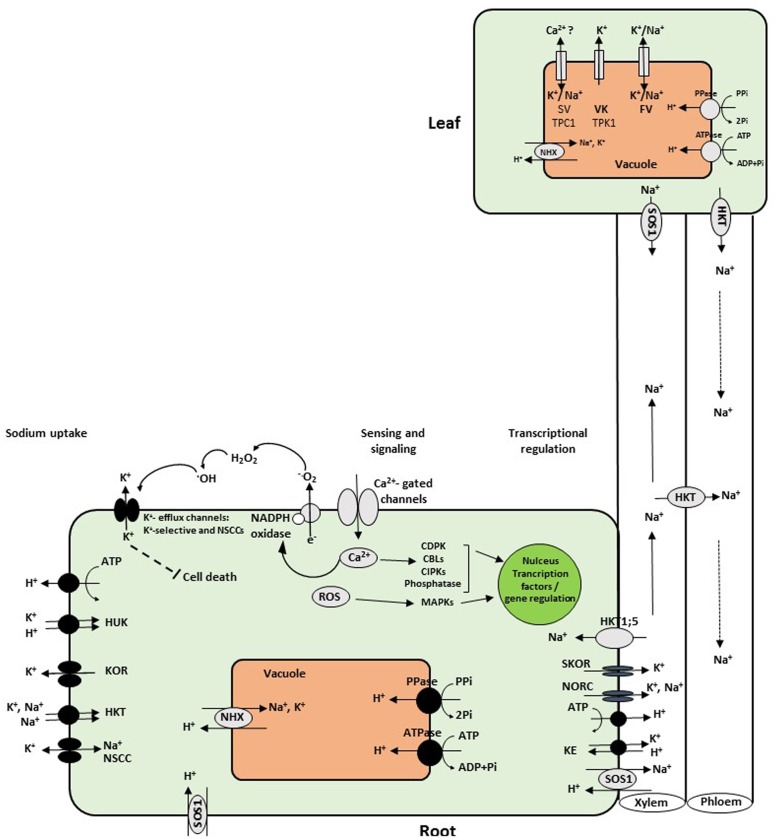
**Schematic overview of sodium uptake into roots and transport mechanisms into leaves.** Na^+^ enters root cells and cross the plasma membrane via NSCC, CNGC, members of the HKT gene subfamily 1, and apoplastic pathways. To cope with salt stress, Na^+^ is sensed by the hyperosmotic and ionic sensors leading to activated Ca^2+^, ROS, and hormone signaling pathways. CDPKs, CBLs, CIPKs, MAPK become active and transduce signal downstream gene transcription in the nucleus. This signaling pathways result in activation of detoxification mechanisms, including the plasma membrane Na^+^/H^+^ antiporter (SOS1), HKT, and the tonoplast Na^+^, K^+^/H^+^ exchanger (NHX). SOS1 extrudes Na^+^ from the cortex cells at the root–soil interface, while at the xylem parenchyma cells; it loads Na^+^ into xylem sap. The HKT1 protein mediates the reverse flux and unloads Na^+^ from the xylem vessels to prevent overaccumulation in photosynthetic tissues. Other candidates for loading Na^+^ to the xylem are the outward-rectifying K^+^ channels KORC and NORC. At the tonoplast membrane, NSCCs include the slow vacuolar (SV) and fast vacuolar (FV) conductances, whereas the vacuolar K^+^ (VK) channel is selective for K^+^, while TPC1 is perfectly leaky for Na^+^ (Maathuis and Amtmann, 1999; Shabala and Cuin, 2007; Kronzucker and Britto, 2011). To maintain low concentration of Na^+^ in leaves, it is either retranslocated with HKT gene through the phloem to lower leaves and down to the roots, or detoxified by sequestration into the vacuole with NHX proteins. NSCCs, nonselective cation channels; HUK, HKT, high potassium affinity transporter; ROS, reactive oxygen species; CDPKs, calcium-dependent protein kinases; CBLs, calcineurin B-like proteins; CIPKs, CBL-interacting protein kinases; MAPK, mitogen-activated protein kinase; KOR, outward-rectifying K^+^channels; NORC, nonselective outward-rectifying channels.

To cope with salt stress, plants have developed the ability to sense both the hyperosmotic and the ionic Na^+^ components of the stress. However, the molecular identities of the plant Na^+^ and hyperosmotic sensors remain unknown until now. Nevertheless, plant hyperosmotic sensors are likely to be coupled with Ca^2+^ channels due to the rapid rise in cytosolic Ca^2+^ levels within seconds of exposure to NaCl or mannitol (Knight et al., 1997; **Figure 1**). The Ca^2+^ signal occurs in roots and in several cell types (Martí et al., 2013). This finding indicates that hyperosmotic stress may be sensed by a mechanically gated Ca^2+^ channel (Kurusu et al., 2013). In a recent study, Choi et al. (2014) suggested that Ca^2+^-dependent signaling plays a role in the process of systemic signaling in response to salt stress. Thus, local salt stress of the root tip leads to the spreading of a Ca^2+^ wave that propagates preferentially through cortical and endodermal cells to distal shoot tissues at speeds of up to 400 μm/s. Salt stress-induced long-distance Ca^2+^ wave is dependent on the activity of the ion channel protein Two Pore Channel 1 (TPC1), which appears to contribute to whole-plant stress tolerance. In *Arabidopsis*, the vacuolar cation channel TPC1 is involved in propagation of calcium waves and mediates the passage of K^+^ and Na^+^. TPC1 plays an important role for cation homeostasis and vacuolar storage function (Larisch et al., 2016). These results suggest that plants do possess a sensory network that uses ion fluxes moving through defined cell types to rapidly transmit information between distant sites within the organism. Downstream of Ca^2+^ signaling, calcium-dependent protein kinases (CDPKs), and calcineurin B-like proteins (CBLs) with CBL-interacting protein kinases (CIPKs) may become active and transduce the hyperosmotic signal to downstream protein activity and gene transcription (Weinl and Kudla, 2008; Boudsocq and Sheen, 2013).

On another hand, potassium as an essential macroelement, is needed at large amounts to be taken up from the soil and transported throughout the plant and enable efficient growth and development (Ahmad and Maathuis, 2014). Under salinity the increase in cytoplasmic Na^+^ and reduction of K^+^ result in changes of membrane potential, osmotic pressure, turgor pressure, calcium signaling, ROS signaling, etc. Recent results on ion fluxes in glycophyte *Arabidopsis thaliana* and the halophytic relative *Thellungiella halophila* showed lower Na^+^ fluxes and higher K^+^/Na^+^ selectivity of ion currents in the roots and root protoplasts of the halophyte under salt treatment (Volkov and Amtmann, 2006; Amtmann, 2009). Maintenance of K^+^ homeostasis is essential for enzyme activities, ionic and pH homeostasis, and cytosolic K^+^ is considered to be an attribute of plant adaptive responses to a broad range of environmental constraints (Shabala and Pottosin, 2014). In addition, a strong correlation between the root’s K^+^ retention ability and plant salinity stress tolerance was reported for several species including wheat (Cuin et al., 2012), barley (Wu et al., 2015), and *Brassica* species (Chakraborty et al., 2016). In fact, this strong correlation between K^+^ retention and net Na^+^ uptake observed in *Brassica* species argue toward involvement of GORK (outward-rectifying potassium selective) channels as a major pathway for the salt stress-induced K^+^ efflux from *Brassica* roots. GORK is central for Na^+^-induced K^+^ loss from root epidermis. GORK channels are activated by membrane depolarization (Véry et al., 2014), and their gating is strongly dependent upon the extracellular K^+^ concentration (Anschütz et al., 2014).

On another hand, it is well established that electrolyte leakage which is considered as a hallmark of plant cell response to abiotic (including salinity) and biotic stresses, is based mainly on K^+^ efflux (Palta et al., 1977). This stress induced K^+^ leakage is often accompanied by ROS generation and leads to cell death (Demidchik et al., 2014). In addition, there are K^+^ outwardly rectifying channels that are induced by ROS and especially hydroxyl radicals (Demidchik et al., 2003, 2010). Therefore, under stress K^+^ leakage, ROS and plant cell-death (PCD) seem to be intimately connected. Such hypothesis was elegantly strengthen by Demidchik et al. (2010) who showed through pharmacological and genetic approaches that blocking K^+^-channel and the lack of functional GORK both inhibit the stress-induced activation proteases and endonucleases that lead to PCD.

### Transport of Sodium and Detoxification Pathways

Na^+^-influx pathways into roots occur via different channels and transporters. Na^+^ may cross the plasma membrane through nutrient channels and calcium-permeable nonselective cation channels (NSCCs), including the cyclic nucleotide-gated channel (CNGC) and the glutamate-like receptor (GLR), which represent a likely entry point of Na^+^ into the cell (Tyerman and Skerrett, 1999; Guo et al., 2008; Deinlein et al., 2014). The role of NSCC is not restricted to mediate Na^+^ influx, which triggers K^+^ efflux via KORG, but they can also contribute to K^+^ efflux directly. Under salt stress and for efficient storage of Na^+^ to the vacuole, NSCC may play important role by preventing Na^+^ leak to the cytosol and without perturbing K^+^ release (Pottosin and Dobrovinskaya, 2014). This leads to the activation of K^+^-efflux channels (GORK) and the loss of K^+^ from the cell, stimulating cell-death enzymes (Demidchik et al., 2010). These functions might be regulated by ROS-activated ion channels in plant roots. Salt-induced K^+^ efflux in relation to Na^+^ influx, which depolarizes the membrane, increasing the driving force for K^+^ efflux and causing the activation of outward-rectifying K^+^ channels (Shabala et al., 2006; Cuin et al., 2008).

The several HKT-type transporters were shown to be involved in Na^+^/K^+^ symport in different plant species including *Arabidopsis*, rice and wheat. Based on sequence and transport analyses, HKT transporters can be classified into two distinct subgroups class I and II, with the first being more Na^+^-selective transport and the second as Na^+^–K^+^ co-transporter (Munns and Tester, 2008; Deinlein et al., 2014). The rice Na^+^ transporter OsHKT2;1 (previously named OsHKT1) has been shown to mediate Na^+^ influx into roots under K^+^ starvation (Horie et al., 2007). Other members of the HKT gene subfamily 1, are thought to mediate Na^+^ influx in root cells and to regulate the Na^+^ distribution between roots and shoots (Sunarpi et al., 2005; Horie et al., 2009).

The Na^+^/H^+^ antiporter SOS1 extrudes Na^+^ from the cortex cells at the root–soil interface, thereby reducing the net uptake of Na^+^. In contrast, at the xylem parenchyma cells, SOS1 loads Na^+^ into xylem sap, whereas HKT1-like proteins mediate the reverse flux and unload Na^+^ from xylem vessels to prevent Na^+^ overaccumulation in photosynthetic tissues (**Figure 1**). Na^+^ enters the xylem by efflux out of stellar cells and is subsequently transported to aerial plant tissues. HKT1-like proteins may facilitate the translocation of Na^+^ to the upper shoot or back to the roots by unloading ions from the xylem and transported to the phloem via symplastic diffusion (Berthomieu et al., 2003; Sunarpi et al., 2005). Studies on xylem parenchyma-localized class I HKT transporters have led to the identification of an essential mechanism for plants to protect photosynthetic organs from Na^+^ overaccumulation and balancing to high K^+^/Na^+^ ratio in plants (Sunarpi et al., 2005). In *Arabidopsis*, the phloem recirculation model proposed by Berthomieu et al. (2003) suggests that Na^+^ is loaded into shoot phloem cells by AtHKT1;1 and then transferred to roots via the downstream phloem flow. In fact, it is generally accepted that AtHKT1;1 mediates the retrieval of Na^+^ from the xylem sap, thereby restricting the amount of Na^+^ reaching the young photosynthetic tissues (Berthomieu et al., 2003; Sunarpi et al., 2005).

Potential candidates for the control of xylem loading of Na^+^ are the outward-rectifying K^+^ channels KORC and NORC (Wegner and de Boer, 1997).

To balance the toxic effect of Na^+^ accumulation and to control ion homeostasis during salinity stress, plants require the maintenance of stable K^+^ acquisition and distribution (Schroeder et al., 1994). The tonoplast-localized Na^+^/H^+^ exchangers (NHX1 and NHX2) and the plasma membrane-localized Na^+^/H^+^ antiporter (SOS1), are thought to play important roles in osmoregulation and to maintain low cytoplasmic Na^+^ concentration in plant cells (Shi et al., 2002; Apse et al., 2003; Brini et al., 2007a; Olias et al., 2009). Most NHX proteins are essential for Na^+^ detoxification through sequestration into the vacuole, whereas SOS signaling pathways were responsible to export Na^+^ outside the cell. The concept that vacuolar NHX proteins were capable of exchanging Na^+^ and H^+^ across the tonoplast as claimed by Apse et al. (1999) and Blumwald (2000), was challenged by the biochemistry of NHX proteins. Indeed, NHX proteins were shown not to discriminate between Na^+^ and K^+^ nor having preference for K^+^ transport (Jiang et al., 2010). Moreover, it has been shown that NHX1 and NHX2 proteins of *Arabidopsis* play a comparatively greater role in K^+^ homeostasis, rather than in Na^+^ sequestration (Leidi et al., 2010; Bassil et al., 2011; Barragan et al., 2012). In addition, NHX1 overexpression in tomato conferred tolerance to NaCl, which was related to the preferential accumulation of K^+^ in vacuoles and improved K^+^ retention after stress imposition, but did not enhance the ability to compartmentalize toxic Na^+^ ions into the vacuole (Leidi et al., 2010).

### Adaptive Mechanisms of Salt Tolerance at the Cell and Organ Level

Research carried out on salinity tolerance mechanisms was mainly performed on model plants (i.e., *A. thaliana*) and only few cases were reported on crop plants. In glycophytes, salinity tolerance is mainly achieved through more than one strategy operating either simultaneously or separately, depending on the duration and intensity of the stress. According to Chakraborty et al. (2016), the overall superior salinity tolerance observed in *Brassica napus* was achieved by the high osmotolerance matched by the moderate tissue tolerance and superior K^+^ retention ability in the leaf mesophyll. Moreover, Chakraborty et al. (2016) provide strong evidence that higher salt tolerance in *B. napus* is conferred by at least three complementary physiological mechanisms: (i) higher Na^+^ extrusion ability from roots correlated with increased expression and activity of plasma membrane SOS1-like Na^+^/H^+^ exchangers; (ii) better root K^+^ retention ability resulting from stress-inducible activation of H^+^-ATPase and the ability to maintain a more negative membrane potential under saline conditions; and (iii) reduced sensitivity of *B. napus* root K^+^-permeable channels to ROS. Shabala et al. (2015) has argued that changes in SOS1 activity strongly correlated with changes in net K^+^ and H^+^ fluxes, making the involvement of the H^+^-ATPase/GORK tandem system as a potential sensor a plausible hypothesis. Therefore, it was suggested that Na+ exclusion from uptake plays an important but not a crucial role as a determinant of genetic variability in salinity stress tolerance in *Brassica*. At the root level, exclusion of ∼95% of salt entering the roots back to the soil solution constitutes the major adaptive trait that plants will undertake to avoid the toxic effect of high salinity in the shoots. Species exhibiting significant genotypic variation in Na^+^ accumulation in leaves have shown a correlation between salt tolerance and Na^+^ exclusion. This is the case in sensitive species like rice and durum wheat (Munns, 2005), but also in more salt-tolerant species like barley (Chen et al., 2005). Indeed, a strong correlation between salt exclusion and salt tolerance does exist in many species (Munns and James, 2003). Further removal of sodium from xylem to translocate back into roots is another way to prevent Na^+^ overaccumulation in photosynthetic tissues. QTL analyses for salinity resistance have suggested that similar xylem Na^+^-unloading mechanisms are essential for salt tolerance in rice and wheat (James et al., 2006). In both cases, major salt tolerance QTL map to regions that include *HKT*1;5 orthologs, encoding a more Na^+^-selective class I HKT transporter (Byrt et al., 2007). When reaching the stem, sodium will either be stored or controlled in its long distance transport. Partitioning of sodium into leaf sheath/petiole during its export through xylem to the leaves, could conceivably help to maintain low salt concentration in the transpiration stream (James et al., 2006; Byrt et al., 2007). However, its retranslocation through the phloem in the lower leaves and down to the roots is considered as relatively limited. To avoid raising cytosolic Na^+^ concentration and balance the toxic effect of Na^+^ accumulation, Garcia de la Garma et al. (2015) reported extensive vesicle trafficking of Na^+^ between the plasma membrane and Na^+^-rich vacuolar compartment in salt-acclimated tobacco BY2 cells. However, and as mentioned above, the enhancement of salt tolerance observed on transgenic tomato plants overexpressing AtNHX1 was related to larger K^+^ vacuolar pools and improved K^+^ retention rather than a compartmentalization of toxic Na^+^ ions into the vacuole (Leidi et al., 2010). The lack of correlation between greater salt tolerance and the enhancement of Na^+^ accumulation in different species overexpressing NHX proteins has been specified by Rodríguez-Rosales et al. (2009) and Jiang et al. (2010). The maintenance of K^+^ acquisition with the exclusion of Na^+^ from photosynthetic leaves has been indeed found to be highly correlated with plant salt tolerance (Hauser and Horie, 2010).

## Strategies to Improve Salt Tolerance in Crops

### Interaction with Beneficial Soil Microorganisms to Improve Salinity Tolerance

Interactions with beneficial soil microorganisms including symbiotic nitrogen-fixing bacteria (*Frankia* and rhizobia) and mycorrhizal fungi can have a large impact on plant tolerance to salt stress. The impact of salt stress on these symbioses has been reviewed elsewhere and will be not covered in this review (Swaraj and Bishnoi, 1999; Zahran, 1999; Serraj and Adu-Gyamfi, 2004; Evelin et al., 2009; Porcel et al., 2012; Ngom et al., 2016b).

Symbiotic associations with arbuscular mycorrhizal fungi (AMF) are found in roughly 80% of terrestrial plant species (Smith and Smith, 2011). These microsymbionts play a critical role in plant nutrition and profit from plant carbon in return (Smith and Read, 2010). In addition, they enhance plant performance and resistance to abiotic stresses (Sadhana, 2014). AMF can alleviate salt stress in host plants by enhancing water absorption capacity, nutrient uptake and accumulation of osmoregulators to increase osmotic potential of cells. Studies have reported that mycorrhizal colonization can reduce the uptake of Cl^-^ ions while preventing Na^+^ translocation to shoot tissues under salinity (Evelin et al., 2009). AMF have been known to occur naturally in saline environments (Evelin et al., 2009). For example, AMF (*Glomus intraradices*, *Glomus versiform*, and *Glomus etunicatum* predominantly) were observed in the severely saline soils of the Tabriz plains of Iran, where soil salinity levels range from 7.3 to 92.0 dS/m (Aliasgharzadeh et al., 2001). The effects of mycorrhizal symbiosis on plant salinity tolerance have been studied in many species including *Medicago sativa* (Azcon and El-Atrash, 1997), *Sesbania aegyptiaca*, and *Sesbania grandiflora* (Giri and Mukerji, 2004), *Z. mays* (Feng et al., 2002; Krishnamoorthy et al., 2016), *Capsicum annum* (Kaya et al., 2009), *Olea europaea* (Porras-Soriano et al., 2009), *Citrus tangerine* (Wu et al., 2010), *Gossypium arboreum* (Tian et al., 2004), and *Lycopersicon esculentum* (Al-Karaki, 2000, 2006; Al-Karaki and Hammad, 2001; Latef and Chaoxing, 2011). In all these species, AMF improved plant salinity tolerance, leading to enhanced plant growth and yield (Azcon and El-Atrash, 1997; Giri and Mukerji, 2004; Diouf et al., 2005; Kaya et al., 2009; Porras-Soriano et al., 2009; Wu et al., 2010), nutrient acquisition (Feng et al., 2002; Giri and Mukerji, 2004; Diouf et al., 2005; Kaya et al., 2009; Porras-Soriano et al., 2009; Wu et al., 2010; Krishnamoorthy et al., 2016), chlorophyll content (Feng et al., 2002; Giri and Mukerji, 2004; Kaya et al., 2009), proline concentration (Diouf et al., 2005; Kaya et al., 2009), and promoting higher accumulation of soluble sugars in roots (Feng et al., 2002; see **Table 1**). In tomato, mycorrhizal colonization significantly improved fruit fresh weight and fruit yield under salt stress (Latef and Chaoxing, 2011). The fruit yield (kg/plant) increased by 33.3 and 106% at 50 and 100 mM salinity levels, respectively. A similar positive effect was reported in the squash *Cucurbita pepo* leading to better fruit yield production in saline conditions (Colla et al., 2008). These benefits of mycorrhizal fungi under saline conditions depend on the symbiotic associations and could therefore be improved by selection of efficient fungal strains.

**Table 1:** Example of beneficial soil microorganisms enhancing plant salinity tolerance.

Beneficial microorganisms inoculum	Plant species	Reference
**Mycorrhizal fungi**		
*Glomus claroideum*	*Olea europaea*	Porras-Soriano et al., 2009
*Glomus clarum*	*Capsicum annum*	Kaya et al., 2009
*Glomus intraradices*	*Acacia auriculiformis*	Diouf et al., 2005
	*A. mangium*	Diouf et al., 2005
	*Cucurbita pepo*	Colla et al., 2008
	*O. europaea*	Porras-Soriano et al., 2009
*Glomus macrocarpum*	*Sesbania aegyptiaca*	Giri and Mukerji, 2004
	*S. grandiflora*	Giri and Mukerji, 2004
*Glomus mosseae*	*Citrus tangerine*	Wu et al., 2010
	*Medicago sativa*	Azcon and El-Atrash, 1997
	*Zea mays*	Feng et al., 2002
	*O. europaea*	Sheng et al., 2008
	*Gossypium arboreum*	Porras-Soriano et al., 2009
	*Lycopersicon esculentum*	Tian et al., 2004
		Al-Karaki, 2000, 2006
		Al-Karaki and Hammad, 2001
		Latef and Chaoxing, 2011
*Paraglomus occultum*	*C. tangerine*	Wu et al., 2010
*Rhizophagus intraradices*	*Zea mays*	Krishnamoorthy et al., 2016
**Rhizobia**		
*Rhizobium* spp. strain AC-2	*A. nilotica*	Bala et al., 1990
*Rhizobium* spp. strain AC-1	A. nilotica	
*Rhizobium* spp. strain L-10	L. leucocephala	
*Rhizobium* spp. strain P-4	*Prosopis juliflora*	
*Rhizobium* PMA63/1	*A. ampliceps*	Zou et al., 1995
*Rhizobium tropici* CIAT899	*Phaseolus vulgaris*	Dardanelli et al., 2008
*Rhizobium etli* ISP42		
*Rhizobium* strain USDA 208	*Glycine max*	Elsheikh and Wood, 1995
*Bradyrhizobium* strain RCR 3407		
***Frankia***		
Crushed nodule suspension	*Casuarina equisetifolia*	Ng, 1987
Crushed nodule suspension	*Alnus glutinosa*	Oliveira et al., 2005
CcI3 strain	*C. equisetifolia*	Ngom et al., 2016a
	*C. glauca*	
CeD strain	*C. equisetifolia*	Ngom et al., 2016a
	*C. glauca*	
**Dual inoculation**		
Mixed spores from *Glomus*, *Gigaspora*, and *Acaulospora genera* + *Sinorhizobium terangae*	*A. saligna*	Soliman et al., 2014
*Glomus mosseae* + *Mesorhizobium mediterraneum*	*Lathyrus sativus*	Jin et al., 2010
*Glomus intraradices* + *Bradyrhizobium* strains Aust 11c and Aust 13c	*A. auriculiformis*	Diouf et al., 2005
	*A. mangium*	
*Glomus clarum* + *Azospirillum brasilense*	*Vigna sinensis*	Rabie et al., 2005
	*Vicia faba*	Rabie and Almadini, 2005
*Rhizophagus intraradices* + *Massilia* sp. RK4	*Zea mays*	Krishnamoorthy et al., 2016
Crushed nodule suspension + *Glomus intraradices*	*A. glutinosa*	Oliveira et al., 2005


Rhizobia are a group of Gram-negative soil heterogeneous bacteria that are also able to form nitrogen-fixing nodules on the roots, or occasionally the shoots, of legumes (Young, 1996) and *Parasponia* species (Akkermans et al., 1978). Bacteria are accommodated intracellularly in nodule cells and fix dinitrogen for plant growth, while being supplied with carbon sources by the plant (Pawlowski and Demchenko, 2012). Many studies have demonstrated that inoculation with suitable *Rhizobium* sp. increase plant dry weight in legumes including *Acacia nilotica*, *Leucaena leucocephala*, *Prosopis juliflora* (Bala et al., 1990), *Acacia ampliceps* (Zou et al., 1995), *Phaseolus vulgaris* (Dardanelli et al., 2008), and soybean (Elsheikh and Wood, 1995) under salt stress. These beneficial effects on plant growth result from an effective N_2_-fixing symbiosis, as acetylene reduction activities were detected even at high salinity levels, depending on the *Rhizobium*–legume associations (Bala et al., 1990; Elsheikh and Wood, 1995; Zou et al., 1995). Indeed, under saline conditions, the salt-tolerant strains of *Rhizobium* sp. formed more effective N_2_-fixing symbiosis with *A. nilotica*, *L. leucocephala*, *P. juliflora* (Bala et al., 1990), *A. ampliceps* (Zou et al., 1995), and soybean (Elsheikh and Wood, 1995) than did the salt-sensitive strains. These results indicate that biological N_2_-fixation under saline conditions may be improved by inoculation with a salt-tolerant *Rhizobium* strain. However, the tolerance of the legume host is the most important factor determining the success of compatible *Rhizobium* strains in forming effective symbioses under conditions of high soil salinity (Craig et al., 1991). Thus, a screening of both symbiotic partners is necessary for obtaining an efficient N_2_-fixing symbiosis under saline soils (Zahran, 1999). Salinity tolerance of legumes could be better improved by associated *Rhizobium* with mycorrhizal fungi (Diouf et al., 2005; Jin et al., 2010; Soliman et al., 2014) and/or plant growth promoting rhizobacteria (PGPR; Rabie and Almadini, 2005; Rabie et al., 2005; Bano and Fatima, 2009). Compared to control treatments, dual inoculation of *Rhizobium* bacteria and mycorrhizal fungi under salt stress, enhanced plant nutrition, growth parameters and proline concentration in *Acacia saligna* (Soliman et al., 2014), *Lathyrus sativus* (Jin et al., 2010), *Acacia auriculiformis*, and *Acacia mangium* (Diouf et al., 2005). Co-inoculation with *Rhizobium* and PGPR including *Azospirillum brasilense* and *Pseudomonas* species showed the same beneficial effects in *Z. mays* (Bano and Fatima, 2009), *Vigna sinensis* (Rabie et al., 2005), and *Vicia faba* (Rabie and Almadini, 2005), which were more pronounced in plant inoculated with AMF, in addition to *Rhizobium* and PGPR (Rabie and Almadini, 2005; Rabie et al., 2005). In Cowpea plants, dual inoculation with AMF and nitrogen-fixing bacteria such as *A. brasilense* increased plant nitrogen content by 230% against 151 and 94% in plants inoculated separately with nitrogen-fixing bacteria and AMF, respectively, at 7.2 dS/m salinity (Rabie et al., 2005).

*Frankia* is a genus of Gram-positive filamentous actinobacteria that can induce the formation of nitrogen-fixing nodulation the roots of 260 species, belonging to eight dicotyledonous families (Betulaceae, Casuarinaceae, Myricaceae, Rosaceae, Eleagnaceae, Rhamnaceae, Datiscaceae, and Coriariaceae), collectively called actinorhizal plants (Benson and Silvester, 1993). Like rhizobia–legume symbioses, bacteria are hosted in root nodules and fix atmospheric nitrogen (Perrine-Walker et al., 2011). Some actinorhizal plants such as Casuarinaceae trees are largely used in land reclamation pros including salinized land (reviewed in Diagne et al., 2013). Several studies have therefore reported strategies to improve the tolerance of these plants to salt stress. Similarly to what occurs in legume–rhizobia symbioses, it has been reported that inoculation with the microsymbiont *Frankia* improves the host plant salinity tolerance (Reddell et al., 1986; Ng, 1987; Oliveira et al., 2005; Ngom et al., 2016a). *Frankia* strains CcI3 and CeD significantly improved *Casuarina glauca* and *Casuarina equisetifolia* plant growth, shoot, root, and total dry weight, proline and chlorophyll contents according to the symbiotic association (Ngom et al., 2016a). According to Ng (1987), inoculated *C. equisetifolia* plants exhibited greater growth (shoot, root, and total dry weight) compared to uninoculated plants under saline conditions. This increase in dry weight was associated with increase in the total nitrogen content of the nodulated plants even at 500 mM NaCl. In saline anthropogenic sediment (conductivity of 5,980 μS/cm), *Alnus glutinosa* plants inoculated with *Frankia* spp. alone significantly increased the growth parameters (total leaf area, shoot height, root collar diameter, and total dry weight), the leaf N content by 197% and the chlorophyll a + b content by 478%, as compared to uninoculated controls (Oliveira et al., 2005). The increased levels of N indicates the effectiveness of the nitrogen fixation process under salinity, and depends on the plant–*Frankia* isolate associations (Reddell et al., 1986). Furthermore, these beneficial effects were significantly greater when *A. glutinosa* plants were inoculated with both *Frankia* spp. and a mycorrhizal fungi, *G. intraradices*. Dual inoculation increased the leaf N, P, and K contents and chlorophyll a + b by 277, 240, 129, and 531%, respectively, suggesting the ability of both microsymbionts in improving actinorhizal plants nutrition under salt-stressed conditions (Oliveira et al., 2005). *Frankia* isolates exhibited diversity in their response to salt stress. Salinity affected their *in vitro* growth and N_2_ fixation depending on the isolate. Among strains studied, *Casuarina* isolates seem to be more tolerant to salinity than others (Ngom et al., 2016b). Under saline conditions, the effects of inoculation of actinorhizal plants by salt-tolerant vs salt-sensitive *Frankia* strains remain poorly studied. Nevertheless, Reddell et al. (1986) showed that under salt stress, *Frankia* ys, collected from *Casuarina obesa* at 1.3 mg Cl^-^/g soil, was able to increase the dry weight of shoot and nodule and the nitrogen content in *C. obesa* plants better than *Frankia* cb, collected from *Casuarina cunninghamiana* at 0.1 mg Cl^-^/g soil. Among three *Frankia* isolates used separately as inoculums of *C. glauca*, Thr which was more *in vitro* sensitive to salt stress, was the most effective strain *in planta*. A recent study showed that inoculation of *C. glauca* plants with the salt-sensitive CcI3 strain improved plants growth under saline conditions while in *C. equisetifolia* plants the salt-tolerant strain CeD was more effective (Ngom et al., 2016a). These results suggest there is no correlation between *in vitro* salt tolerance of *Frankia* strains and their effectiveness in association with plants under salt stressed conditions (Girgis et al., 1992; Ngom et al., 2016a). Thus, as what was observed in *Rhizobium*–legumes symbiosis, effectiveness of established actinorhizal symbioses to saline conditions is primary dependent on the salt tolerance of the host plant (Girgis et al., 1992).

Taken together, these studies indicate that AMF and nitrogen-fixing bacteria could be used to increase salt tolerance both in crops and in plants used for saline soils rehabilitation. Co-inoculation with different beneficial microbes has the potential to further increase tolerance. These beneficial microbes improve plant growth and nutrition and promote higher accumulation of osmolytes such as proline and sugars in saline environments. This leads to higher crop yield but also to products with better nutritional properties (Latef and Chaoxing, 2011). To fully exploit the beneficial effect of these symbioses, they need to be taken into account in breeding programs for salt-resistant crop varieties. Breeder will need to identify QTLs that control the response of the plant to these beneficial microbes in normal and salt-stress conditions. These QTLs can then be included in breeding programs along more classical salt-resistance QTLs. Furthermore, the development of industrial scale microbial inoculants for salinized soils will be required.

### Salt Tolerance in Crops through Marker-Assisted Selection and Genetic Engineering

Conventional plant breeding approaches through which beneficial traits can be introgressed into elite varieties have been adopted since a long time to generate stress tolerant varieties (for review, see Ashraf, 2010; Varshney et al., 2011). Hence, traditional breeding allowed the development of new salt-tolerant rice and wheat varieties (Munns et al., 2006; Singh et al., 2010). However, as salinity tolerance is a multigenic trait, such approaches have only limited success, which can explain the absence of commercially available salt-tolerant crops. Moreover, as a procedure, plant breeding is time consuming and labor intensive, relies on well characterized germplasms and can result in introducing undesirable traits along with the selected one. Therefore, biotechnological approaches including molecular breeding and genetic engineering seem to be more attractive alternatives.

#### Plant Salinity Tolerance through Genetic Engineering

In the last 30 years, tremendous progress has been made toward the isolation and molecular characterization of genes involved in plant salt stress responses. These candidate genes can be classified into two main groups: effectors and regulatory genes. The first group includes mainly genes encoding ion transporters, channels, enzymes involved in osmolyte biosynthesis, antioxidant systems and protective proteins such as heat shock proteins and late embryogenesis abundant (LEA) proteins. The second group is composed of genes involved in transcriptional and post-transcriptional regulation as well as in signaling pathways. Within this group we find essentially transcription factors, protein kinases, phosphatases, and proteases. Numerous reports described how the overexpression of these genes can improve plant tolerance to various abiotic stresses including salinity (for review, see Ashraf, 2009; Türkan and Demiral, 2009; Cominelli et al., 2013; Roy et al., 2014; Fita et al., 2015; see **Table 2**).

**Table 2:** Example of genes leading to improvement of salt stress tolerance of crop plants through genetic engineering.

Transgene	Function	Donor	Transgenic plant	Description	Reference
Na^+^/H^+^ antiporter (*AtNHX1*)	Vacuolar sequestration of Na^+^ and K^+^?	*Arabidopsis*	Tomato	Enhanced salt tolerance with higher accumulation in leaves but not in fruits	Zhang and Blumwald, 2001
			*Brassica napus*	Maintenance of seed yield and seed oil quality under high salinity	Zhang et al., 2001
			Wheat	Improved grain yield in saline soils	Xue et al., 2004
			Cotton	Increased fiber yield under salt stress	He et al., 2005
H^+^-pyrophosphatase (*AVP1*) *AtNHX* + *AVP1*	Vacuolar membrane-bound proton pump	*Arabidopsis*	Cotton	Increased fiber yield under salt stress in field conditions	Pasapula et al., 2011
			Barley	Higher biomass production and grain yield in saline field	Schilling et al., 2013
			Cotton	Further enhancement of salt tolerance compared to single-gene overexpressing plants	Shen et al., 2014
			Tomato	Increased salt stress tolerance compared to single gene overexpression	Gouiaa and Khoudi, 2015
*Ncl*	homologous to NHX gene family	Soybean	Soybean	Improved salt tolerance	Do et al., 2016
Mannitol-1-phosphate dehydrogenase *mt1D*	Mannitol biosynthesis	*E. coli*	Tobacco	Increased salt tolerance	Tarczynski et al., 1992
Choline synthase *codA*	Betaine biosynthesis	*E. coli*	Rice	Enhanced tolerance to salinity and cold	Sakamoto et al., 1998
delta 1-pyrroline-5-carboxylate synthase (*P5CS*)	Proline biosynthesis	*Arabidopsis*	Tobacco	Increased tolerance to drought and salt stress	Kishor et al., 1995
ascorbate peroxidase (*AtAPX*)	ROS-scavenging	*Arabidopsis*	Tobacco	Enhanced tolerance to salt and osmotic stress	Badawi et al., 2004
Late embryogenesis abundant protein (*HVA7*)	Osmoprotection	Barley	Rice	Enhanced tolerance to salt and osmotic stress	Xu et al., 1996
Transcription factor *DREB1B/CBF1*	Transcription regulation	*Arabidopsis*	Rice	Enhanced tolerance to salinity and drought	Oh et al., 2005
Transcription factor *DREB1A/CBF3*	Transcription regulation	*Arabidopsis*	Rice	Increase in salinity tolerance	Hu et al., 2006
*SNAC1*	Transcription regulation	Rice	Rice	Increase in salinity tolerance	Hu et al., 2008.
Transcription factor *SlAREB1*	Transcription regulation	*Tomato*	Tomato	Enhanced tolerance to salinity and water stress	Orellana et al., 2010
Transcription factor *ZFP179*	Transcription regulation	Rice	Rice	Increase in salinity tolerance	Sun et al., 2010
calcium-dependent protein kinase *OsCDPK21*	Calcium signaling	Rice	Rice	Increase in salinity tolerance	Asano et al., 2011
MAP kinase *GhMPK2*	MAPK signaling	Cotton	Tobacco	Enhanced tolerance to salinity and drought	Zhang et al., 2011


##### Ion transporters

Ion transporters are obvious candidates among effectors since they participate in salt detoxification and ion homeostasis. Overexpression of genes involved in Na^+^ transport was therefore largely investigated. The *Arabidopsis* vacuolar Na^+^/H^+^ antiporter (AtNHX1) was among the first candidate genes which when overexpressed lead to enhanced salinity tolerance (Apse et al., 1999). These transgenic plants were reported to exhibit higher ability for vacuolar sequestration of Na^+^ to avoid its toxic accumulation into the cytoplasm. Later on, overexpression of AtNHX1 and related NHX proteins from various sources have been shown to increase salt tolerance in other plant species including tomato (Zhang and Blumwald, 2001), *B. napus*, wheat and cotton (Pardo et al., 2006; Munns and Tester, 2008). Interestingly, the tomato AtNHX1 overexpressing plants are more salt stress tolerant, with an accumulation of NaCl in leaves but not in fruits. However, and as was indicated above, its role in salt stress tolerance may also result from its capacity to facilitate K^+^ uptake at the tonoplast (Leidi et al., 2010). Nevertheless, engineering of vacuolar cation/H^+^ antiporter coupled with its H^+^-translocating pyrophosphatase (H^+^-PPase) often leads to increased salt stress tolerance. Therefore, overexpression of the wheat TNHX1 and TVP1 improves salt stress tolerance in *Arabidopsis* (Brini et al., 2007a), tobacco (Gouiaa et al., 2012), and tomato (Gouiaa and Khoudi, 2015). Alone the *Arabidopsis* H+-PPase (AtAVP1) was also demonstrated to increase stress tolerance in a crop plant. Indeed, Schilling et al. (2013) reported that barley transgenic plants overexpressing AtAVP1 are more tolerant to salinity under greenhouse conditions but they also showed increased shoot biomass production and grain yield in a saline field.

On another hand, the HKT family and the SOS pathway play also a relevant role in controlling Na^+^ transport within the plant and have been considered in transgenic approaches to increase salt stress tolerance in crops. However, knowing their role in the removal of Na^+^ from the xylem sap into the surrounding xylem parenchyma cells, improving salinity tolerance using HKTs can be successful only when its expression is targeted to the stele or driven by salt-inducible promoter (Møller et al., 2009; Roy et al., 2014). Recently, the overexpression of *Ncl* gene (homologous to the Na^+^/H^+^ antiporter gene family) into a Japanese soybean salt-sensitive cultivar Kariyutaka, resulted in improved salt tolerance in transgenic soybean. A close association was observed between the high expression of the salt tolerance gene *Ncl* in the root, the lower accumulation of Na^+^, K^+^, and Cl^-^ in the shoot under salt stress and salt tolerance in the transgenic lines (Do et al., 2016).

##### Osmolyte accumulation

Under salt stress, along with Na^+^ exclusion from the cytoplasm, plant cell accumulates a wide range of compatible solutes or osmolytes to balance the osmotic pressure of ions in vacuoles. Sucrose, proline, and glycine betaine are among the most studied osmolytes accumulating upon salt stress in some plant species including halophytes (Flowers et al., 1977). Therefore, plant engineering for higher accumulation of these compounds was considered as a possible way in improving crop tolerance to salinity. The bacterial *mt1D* gene encoding the mannitol-1-phosphate dehydrogenase, enzyme involved in mannitol biosynthesis was, early on, successful in enhancing salt tolerance after its ectopic expression in tobacco (Tarczynski et al., 1992, 1993). Similarly, transgenic rice overexpressing choline oxidase showed increased levels of glycine betaine and enhanced tolerance to salinity and cold (Sakamoto et al., 1998). Also, plants such as tobacco transformed with delta 1-pyrroline-5-carboxylate synthase (P5CS) gene exhibited higher proline production, correlated with increased tolerance to drought and salt stress (Kishor et al., 1995). Similarly, transgenic rice plants expressing the mothbean P5CS gene under constitutive or stress inducible promoter showed significant tolerance to high levels of NaCl (Su and Wu, 2004).

##### Antioxidant systems and protective proteins

Despite their importance as signaling molecules regulating cellular responses to various stresses (for review, see Apel and Hirt, 2004), ROS can also damage plant tissues during salinity stress by perturbing enzyme, cell wall and membrane function. Plants detoxify then ROS generated by salt stress by up-regulating antioxidative enzymes such as SOD, CAT, APX, and glutathione peroxidase. Therefore, overexpressing ROS-scavenging enzymes were shown to promote tolerance of plants to various stresses including salinity (Rodriguez-Rosales et al., 1997; Roxas et al., 1997; McKersie et al., 1999; Badawi et al., 2004; Miller et al., 2008). For example, the overexpression of ascorbate peroxidase in tobacco chloroplasts enhances the tolerance to salt stress and water deficit (Badawi et al., 2004).

Other proteins like osmotin and LEA proteins contribute in alleviating salt stress by protecting macromolecules from damages caused by ion toxicity and/or water deficit. HVA7, a LEA from barley, when transferred to rice, confers water and salt stress tolerance (Xu et al., 1996). It is worth to note that, among the different groups, the group 2 of LEA proteins known as dehydrins are particularly interesting and were shown to enhance plant tolerance to various stresses (Hanin et al., 2011). Indeed, Brini et al. (2007b) showed that the expression of the wheat dehydrin DHN-5 in *A. thaliana* led to an increase in salt and osmotic stress tolerance, but also evidence is provided for the involvement of DHN-5 in other abiotic and biotic stress responses (Brini et al., 2011; Drira et al., 2015).

##### Transcription factors and signaling proteins

Plant response to salinity is complex and involves multiple genes involved in distinct or overlapping regulatory pathways. Therefore, the engineering of a single downstream effector gene as indicated above, albeit efficient in some circumstances might have limited success when one considers multiple stress combination as occurring in the field. In contrast, regulatory proteins such as transcription factors and signaling proteins will gain increasing interest as they are expected to modulate the expression of numerous downstream genes involved in stress responses. It is well documented that transcription factors belonging to the families of DREB, NAC, MYB, MYC, Cys2/His2 zinc finger, bZIP, AP2/ERF, and WRKY are relevant in salt stress tolerance (Golldack et al., 2011, 2014).

In this regard, several transcription factors such as DREBs, MYCs, AP2/ERFs, and NACs were tested in model plant species and few crops. In some cases, the overexpression of these transcription factors was successful to enhance salinity tolerance in crops (for review, see Lata and Prasad, 2011; Lata et al., 2011; Turan et al., 2012; Nuruzzaman et al., 2013).

The expression of DREB1B/CBF1 or DREB1A/CBF3 under the control of the cauliflower mosaic virus 35S promoter in *Arabidopsis* plants increases significantly tolerance to freezing, drought, and high salinity stresses (Jaglo-Ottosen et al., 1998; Liu et al., 1998; Kasuga et al., 1999). Similarly, transgenic rice plants constitutively expressing DREB1A/CBF3 were reported to be more tolerant to drought and salinity (Oh et al., 2005). Also, the overexpression of SNAC1 or SNAC2 (stress responsive NAC) resulted in an enhanced salinity tolerance (Hu et al., 2006, 2008).

SlAREB1 is a bZIP transcription factor from tomato (*Solanum lycopersicum*), member of the ABA-responsive element binding protein (AREB)/ABA-responsive element binding factor (ABF) subfamily. Its overexpression in tomato was reported to improve tolerance to water and salt stresses (Orellana et al., 2010). Likewise, Sun et al. (2010) have shown that the overexpression of ZFP179, a salt responsive gene encoding a Cys2/His2 zinc finger protein enhanced salt tolerance in rice.

Using strong and constitutive promoters to drive the expression of transcription factors is still, however, considered as a controversial strategy. The constitutive expression of transcription factors caused in some cases growth defects under standard conditions as was reported for the 35S:TaDREB1 rice transgenic plants overexpressing the bread wheat DREB1 gene that showed a dwarf phenotype (Shen et al., 2003). Therefore, one should reconsider the use of constitutive promoters and rather employ stress inducible/tissue-specific promoters to avoid secondary deleterious effects (Munns and Tester, 2008).

Moreover, effects of the overexpression of genes encoding signaling proteins such as kinases and phosphatases on salt tolerance were reported. As conserved signaling proteins at the crossroads of several signaling cascades, MAPKs play pivotal role in plant responses to various stresses. Many transgenic plants that have been engineered with MAPK cascade were reported to be tolerant to salt stress. The ectopic expression of cotton *GhMPK2* improves salinity and drought tolerance in tobacco (Zhang et al., 2011). In addition, CDPKs, which are involved in salt stress response, were found efficient in transgenesis approaches, as was reported for the overexpression of *OsCDPK21* that resulted in increased salinity tolerance of transgenic rice (Asano et al., 2011). However, other MAPKs can have opposite effects and this was described for the rice OsMAPK33, the overexpression of which caused higher sensitivity to drought and salinity compared to wild-type plants (Lee et al., 2011). Regulators of MAPKs, the MAPK phosphatases (MKPs) can also be involved in the control of stress responses. Interestingly, while the *Arabidopsis* AtMKP1 acts as a negative regulator, the wheat counterpart acts as a positive regulator of salt stress responses (Ulm et al., 2002; Zaidi et al., 2016).

Noteworthy, it is still until now problematic to generate and commercialize crops more tolerant to salinity or to any other stresses. This can be due to several factors. First of all, the laboratory growth conditions are far different from field conditions. Field tests are often forbidden or restricted in several countries but mainly one needs to consider the reproducibility of the field tests and be able to measure and monitor any environmental changes that may affect crop yield (humidity, soil composition, light intensity,…). Second, often the stress tolerance of transgenic crop grown in a greenhouse or a growth chamber is assessed by measuring survival and or recovery rates, while for farmers; the main trait for stress tolerance is crop yield. Moreover, in the first case the evaluation of stress tolerance level is limited to particular traits (plant height, leaf or root size) or development stages during the vegetative phase and only occasionally covers the reproductive stage. The need to conduct field trials under monitored conditions over several years in distinct environmental conditions is a necessary condition to ensure the development of sustainable genetically improved salt-tolerant crops.

#### Marker-Assisted Selection and Salt Stress Tolerance

One of the major limitations for the use of conventional breeding to improve salt tolerance in crops is its slowness which is closely linked to the complexity of this polygenic trait. Traditionally, the selection of crops raised from backcrosses of genetically diverse germplasms is limited to their phenotype analyses in the field. As an alternative to streamline this process, breeders use QTL analyses coupled with marker-assisted selection (MAS), referred as an approach linking a quantitative trait with a genetic marker that is polymorphic between parental lines (Ashraf and Foolad, 2013). Thus still, accumulating knowledge on plant salt stress response is a must to be able to develop confident and efficient markers. The fact that maize, rice, barley, sorghum, and soybean genomes are sequenced and the advances made in next generation sequencing (NGS) helps nowadays to develop high-resolution genetic maps to produce salt-tolerant crops. A quick search on ene database for QTL related to salt revealed 17 QTLs in *Oryza sativa* covering six chromosomes and delimited by several markers^2^. “Saltol” was reported as a promising QTL for salinity tolerance in rice that was identified after searching for more than 100 SSR markers in 140 recombinant inbred lines between Pokkali and IR29 (Thomson et al., 2010). “Saltol” is located on chromosome 1 and is important for the maintenance of the shoot ratio of Na^+^/K^+^ (Thomson et al., 2010). Other QTL analyses performed in wheat for Na^+^ tolerance allowed the identification of Nax1 locus, which maps to the region of the TaHKT1;4 gene that contributes to Na^+^ removal from xylem in the leaf sheath avoiding its over-accumulation in leaf blades (Huang et al., 2006). In soybean, genetic variation for salt tolerance has been described and was observed in wild and cultivated soybean species, suggesting that genetic improvement of salt tolerance is feasible (Lee et al., 2008; Qi et al., 2014). A major QTL for salt tolerance was constantly detected on soybean chromosome 3 (linkage group N) in different populations (Lee et al., 2004; Hamwieh et al., 2011). This QTL is likely to be the *Ncl* locus based on pedigree tracing (Lee et al., 2004). Recently, the introgression of the tolerance allele *Ncl* into soybean cultivar Jackson, using DNA MAS, produced an improved salt-tolerant line (Do et al., 2016).

Despite these successful examples, MAS based breeding pros are still limited because undesirable traits may be transferred with the QTL when wild relatives are used as donors and the results raised from QTL analyses must be confirmed in different conditions and genetic backgrounds. In fact, the cultivated soybean germplasm may be more efficiently used in breeding cultivars with improved salt tolerance compared with that of wild soybean, which generally possesses several undesirable agronomic traits.

Nevertheless, NGS technologies will significantly contribute into discovering molecular markers to obtain high density genetic maps, a prerequisite for a precise location and quicker cloning of new QTLs. Moreover, the advent of genomic selection should speed up the production of varieties combining several salt-resistance QTLs.

## Conclusion

Over the last two decades, research made on *Arabidopsis* and a few crops shed light on several aspects of the molecular mechanisms controlling the salt stress tolerance. However, many challenges still lie ahead before successfully improving crop yield under saline conditions. Hopefully, available tools including molecular breeding and advanced biotechnology methods combined to the exploitation of the potential of soil microorganisms can speed up the release of salt-tolerant crop varieties. A combination of approaches will accelerate the identification and characterization of specific loci involved in tolerance to salinity that can be introgressed into elite sensitive varieties through molecular marker-assisted breeding. To achieve this goal, we need to have diversity in germplasm resources, high-throughput phenotyping platforms, genome sequencing of crops and their relatives. Furthermore, molecular genetic resources including mutation detection, gene discovery and expression profile, genome wide association studies, and powerful omics databases are also needed. Many traits related to salt stress tolerance have been identified and shown effective for engineering stress tolerance in model plants. The most impressive results were obtained when manipulating signaling factors, as they control a broad range of downstream events, which results in superior tolerance. Effective expression systems, including cell type-specific and stress-inducible promoters will be required to adapt the plant response to stress, lower the energy cost, according to the environmental constraints as using constitutive promoters can have severe drawbacks on plant growth or yield. Finally, the targeted genome editing using CRISPR-Cas9 technology has emerged as an alternative to classical plant breeding and transgenic (GMO) methods. CRISPR-Cas9 technology enables precision design of alleles that aid stress tolerance (Zhang H. et al., 2014), but in depth study of genome editing to engineer mechanisms of salt stress tolerance needs to be pursued in the coming years.

## Author Contributions

KM handled the entire process of preparing this paper and prepared the topic on salt tolerance mechanisms in plants. MH and CE prepared the topics related to salinization in arid and semi arid region and the problem of land degradation, the impact of soil salinization on plant growth and survival and salt tolerance in crops through marker-assisted selection and genetic engineering. Finally, LL and MN prepared the topic related to the interaction with beneficial soil microorganisms to improve salinity tolerance. All authors reviewed the manuscript.

## Conflict of Interest Statement

The authors declare that the research was conducted in the absence of any commercial or financial relationships that could be construed as a potential conflict of interest.

## Footnotes

1 https://www.un.org/development/desa/en/news/population/2015-report.html

2 http://ene.org
